# Machine learning–based quantification of overall and internal ultrasound characteristics for diagnosing malignant partially cystic thyroid nodules

**DOI:** 10.3389/fendo.2025.1635122

**Published:** 2025-08-06

**Authors:** Yutong Zhang, Jue Jiang, Aqian Chen, Dong Zhang, Lirong Wang, Xin Yuan, Xin He, Shanshan Yu, Juan Wang, Qi Zhou

**Affiliations:** ^1^ Department of Ultrasound, The Second Affiliated Hospital of Xi ‘an Jiaotong University, Xi’an, China; ^2^ National Key Laboratory of Human-Machine Hybrid Augmented Intelligence, National Engineering Research Center for Visual Information and Applications, and Institute of Artificial Intelligence and Robotics, Xi’an Jiaotong University, Xi’an, China

**Keywords:** partially cystic thyroid nodules, machine learning, ultrasound-based quantification, diagnostic modeling, internal and overall sonographic characteristics

## Abstract

**Introduction:**

Partially cystic thyroid nodules (PCTNs) with malignant potential are frequently underestimated due to limited recognition of their sonographic characteristics.

**Methods:**

This retrospective analysis included 486 PCTNs identified between March 2021 and September 2022. Machine learning (ML) was employed to quantitatively evaluate the overall ultrasound characteristics of the whole nodule as well as the internal ultrasound characteristics of its solid part. Three diagnostic models were constructed based on different sets of ultrasound data. The dataset was split into training and testing subsets at a 7:3 ratio. Key ultrasound characteristics such as marked hypoechogenicity, calcifications, solid component≥50%, and unclear internal margins were emphasized.

**Results:**

Among the models, the integrated one— incorporating both overall-nodule and internal solid-part characteristics—achieved superior diagnostic performance, with an area under the curve (AUC) of 0.96 (0.93-0.99) on the test data. The model demonstrated an accuracy of 0.91 (0.85-0.95), a sensitivity of 0.88 (0.73-0.97), a specificity of 0.92 (0.85-0.96), a negative predictive value of 0.96 (0.91-0.99), and a positive predictive value of 0.77 (0.61-0.89). This comprehensive model significantly outperformed the model utilizing only overall nodule characteristics (AUC = 0.85, P = 2.35e-6), and demonstrated comparable effectiveness to the model based solely on internal characteristics (AUC = 0.93, P = 1.01e-1).

**Discussion:**

The results support the clinical utility of an ML-driven approach that integrates comprehensive ultrasound metrics for the reliable identification of malignant PCTNs.

## Introduction

Improvements in ultrasound device resolution have led to a progressive yearly increase in the detection of thyroid nodules (1, 2). Consequently, partially cystic thyroid nodules (PCTNs) are being identified with greater frequency, representing approximately 27.0% of all detected thyroid nodules (3). PCTNs are defined as nodules with a cystic-solid composition, where the cystic component ranges from 5% to 95% (4). Currently, an increasing number of malignant PCTNs are being detected, representing 3.3% to 17.6% of all cases (5). Despite increasing detection, many PCTNs continue to be classified as having a low malignancy risk, with the likelihood estimated at 2%–5% according to the American College of Radiology Thyroid Imaging Reporting and Data System (ACR-TIRADS) (6, 7).One of the primary contributors to misdiagnosis is the limited awareness among clinicians regarding the ultrasound characteristics associated with malignant PCTNs. Such diagnostic oversights may result in local invasion and lymphatic metastasis (8, 9). Therefore, improving physicians’ comprehension and interpretation of the ultrasound characteristics of PCTNs is crucial for enhancing diagnostic accuracy and facilitating more reliable differentiation between benign and malignant nodules.

Ultrasound risk characteristics outlined in the ACR-TIRADS guidelines—such as a taller-than-wide shape, irregular and unclear margins, microcalcifications, and hypoechoic solid components—have been investigated for their diagnostic utility in distinguishing malignant PCTNs from benign ones (5, 10). However, these characteristics have shown limited diagnostic value, with the area under curve (AUC) values ranging from 0.50 to 0.85 (10). To address these limitations, some researchers have attempted to use machine learning (ML) classification methods to intelligently extract deeper features of PCTNs (11, 12). This approach holds promise not only for identifying more diagnostically relevant characteristics to enhance the prediction of malignant PCTNs, but also for mitigating misdiagnoses that arise from limited subjective clinical experience.

Although machine learning (ML) techniques have been applied by researchers to improve the predictive accuracy for malignant PCTNs, the reported area under the curve (AUC) values have ranged from 0.824 to 0.909 (12). We believe that, for PCTNs, greater attention to the internal ultrasound characteristics of the nodules may be of particular importance. At the same time, most existing ML-based models function as black boxes, providing binary classifications of PCTNs as malignant or benign without offering interpretable clinical information—such as aspect ratio, boundary regularity, or the presence of microcalcifications. Due to their lack of transparency, such models are often met with skepticism and are less readily adopted in clinical settings. In our preliminary investigation, we developed an interpretable ML model capable of generating risk-related ultrasound characteristics of thyroid nodules and presenting them in a clinically meaningful textual format (13). This strategy assists physicians in the precise identification of nodular characteristics. Nonetheless, the potential of such models in predicting malignant PCTNs has not been widely recognized. Accordingly, the present study aims to construct specialized and interpretable ML models capable of quantifying the ultrasound risk characteristics of PCTNs and delivering these outputs in a clinically relevant and readily comprehensible format for healthcare professionals.

## Patients and methods

### Patients

The study was approved by the Medical Ethics Committee of the Second Affiliated Hospital of Xi’an Jiaotong University (IRB number 2022259). All experimental procedures were conducted in compliance with applicable guidelines and regulatory standards, with informed consent obtained from all participants prior to their inclusion in the study.

**Figure 1:**
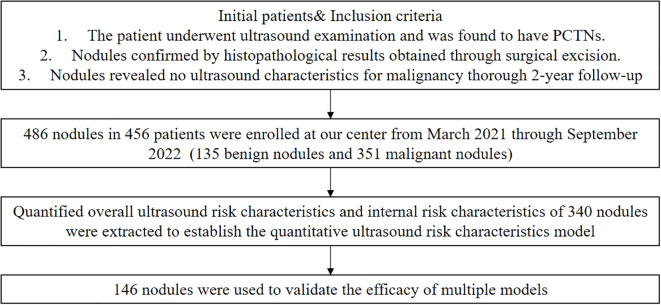
Flowchart of recruiting patients with PCTNs in this study.

We collected ultrasound-confirmed PCTNs at the Second Affiliated Hospital of Xi’an Jiaotong University between March 2021 and September 2022. The inclusion criteria were: 1) nodules confirmed by histopathological results obtained through surgical excision within a six-month follow-up, and 2) for nodules without pathological results, a thorough 2-year follow-up revealed no ultrasound characteristics suspicious for malignancy. A total of 500 nodules were initially included in the study. However, 12 nodules were excluded due to poor ultrasound image quality, and 2 nodules were excluded because they had undergone previous surgical operations or ablation treatments before the ultrasound examination. Ultimately, 486 PCTNs from 456 patients were included, comprising 351 benign and 135 malignant nodules. The flowchart is shown in **Figure 1**.

### Identification of ultrasound characteristics in PCTNs

In this study, thyroid nodules images were collected using various ultrasound diagnostic machines, including Hitachi Hi Vision Ascendus, ACUSON Sequoia, and Logiq E9, all equipped with high-frequency superficial probes ranging from 5 to 18 HZ.

In accordance with ACR-TIRADS recommendations, two experienced physicians (with 7 and 10 years of expertise in thyroid ultrasound diagnosis, respectively) independently re-evaluated and recorded the overall ultrasound characteristics of each nodule. Each physician annotated a subset of the cases. Evaluated characteristics comprised aspect ratio (taller-than-wide or wider-than-tall), echogenicity (hypoechoic, isoechoic, or hyperechoic), margin appearance (irregular or smooth), and the presence or absence of both microcalcifications and macrocalcifications. Subsequently, the internal ultrasound characteristics of the solid component within each PCTN were further assessed and recorded. These characteristics included the proportion of the solid part in relation to the entire nodule (categorized as ≥50% or <50%), the clarity of the solid component’s margin (classified as clear or unclear), and the presence or absence of an eccentric configuration. A PCTN was defined as exhibiting an eccentric configuration if the predominant cystic or solid component was situated at the periphery of the nodule (14). Additionally, the angle between the solid component and the adjacent cyst wall was assessed, and each nodule was categorized as exhibiting either an acute or blunt angle. Representative ultrasound images illustrating these characteristics are provided in **Supplementary Figure 1**.

In cases where the annotating physician was uncertain about a characteristic, the two physicians discussed the case to reach a consensus. If uncertainty remained, a senior physician with 17 years of thyroid ultrasound experience was consulted for the final decision. The original data supporting this study is openly available on GitHub: https://github.com/ZDongLab/ultrasoundimages2d.

### Image preprocessing and segmentation of thyroid nodules in ultrasound imaging

**Figure 2:**
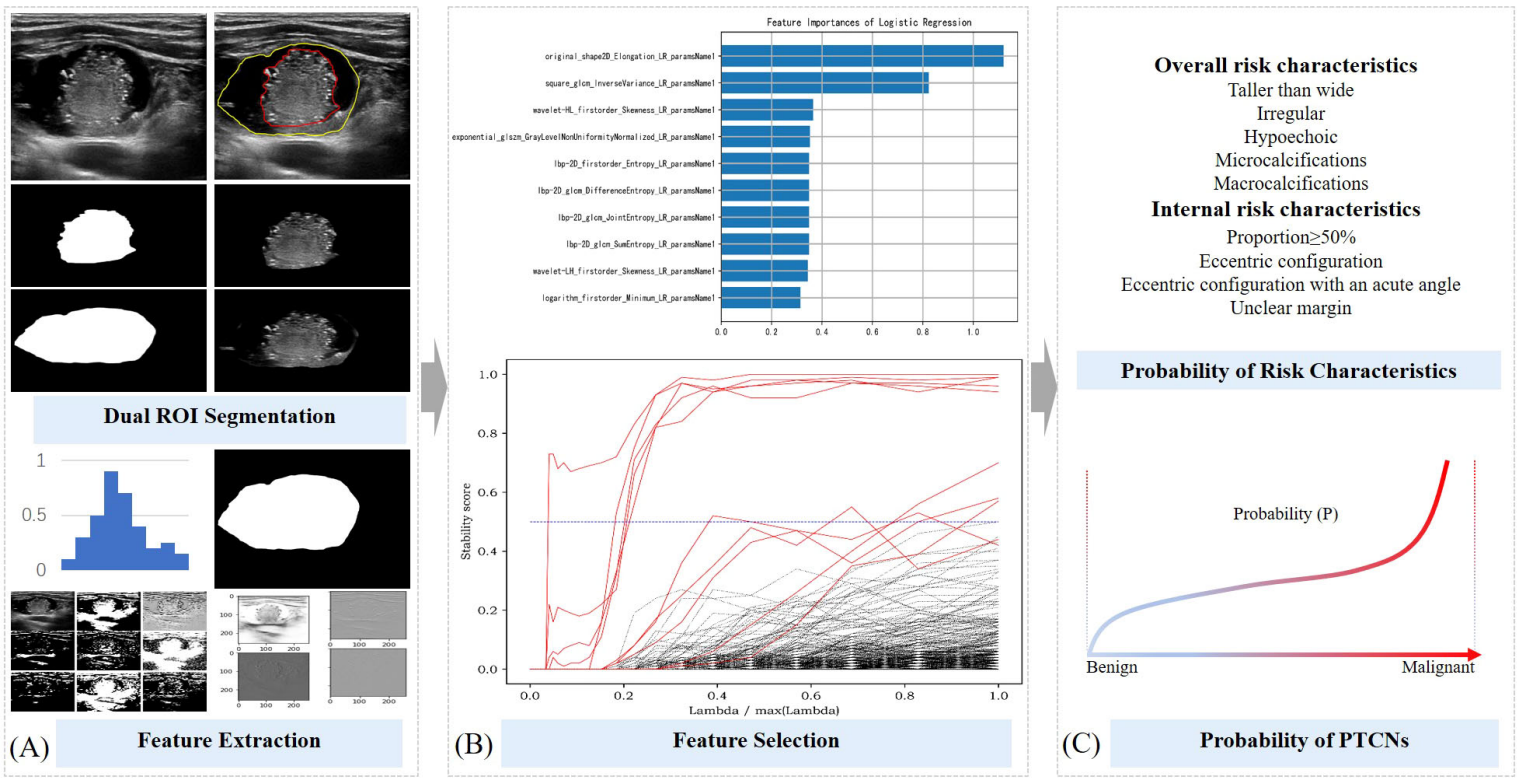
Schematic diagram of ML probabilistic models of risk characteristics in PCTNs. **(A)** Segmentation of ROI and feature extraction; **(B)** Feature selection process; **(C)** Calculation of risk probability for each feature and for the entire nodule.

The original grayscale ultrasound images of PTCNs were imported into the Darwin scientific research platform ( *http://premium.darwin.yizhun-ai.com* ). Regions unrelated to thyroid anatomy, such as black areas lacking tissue signal, were automatically excluded during image preprocessing. Using ITK-SNAP software, two experienced thyroid ultrasound physicians manually delineated the contours of both the entire nodule and its solid component. These annotated and preprocessed images were used to extract and preserve the corresponding regions of interest (ROIs), which were subsequently processed by a segmentation network to segment the two defined areas. (Shown in **Figure 2A**).

### Feature extraction and selection for ultrasound-based analysis of PCTNs

The two segmented image components were processed using a classification network available on the Darwin scientific research platform, enabling the model to learn features associated with five overall ultrasound risk characteristics of the whole nodule and four internal characteristics pertaining to its solid portion in PCTNs. From this process, 1,125 features were initially extracted. To ensure robust feature selection while mitigating overfitting and multicollinearity, we employed a stability-based approach that evaluated feature importance consistency across multiple data partitions. Features demonstrating high predictive stability were retained, while redundant or noisy features were naturally excluded through this process. This resulted in a refined subset of 10 to 22 highly discriminative features for final model training (Shown in **Figure 2B**). Detailed results of the feature extraction and selection are provided in **Supplementary Table 1**.

### Development of machine learning models for predicting ultrasound risk characteristics in PCTNs

The 486 PTCNs were randomly divided into a training set and a testing set at a 7:3 ratio, resulting in 340 nodules for training and 146 nodules for independent testing (Shown in **Figure 2C**). The selected features were then used to develop predictive models estimating the probability of five overall risk characteristics of the entire nodule and four internal risk characteristics of its solid portion. Four commonly used machine learning classifiers—support vector machine (SVM), random forest (RF), logistic regression (LR), and gradient boosting decision tree (GBDT) —were initially evaluated during model development. After comparative analysis, logistic regression classifiers with L2 regularization (ridge regression) were ultimately selected for all risk characteristics, balancing model interpretability and predictive performance. Hyperparameter optimization, including the regularization strength, was performed via cross-validation.

The final predictive formulas for each ultrasound risk characteristic are provided in **Supplementary Table 1**. Model performance was evaluated on the independent testing set of 146 nodules.

### Integrative modeling of overall and internal ultrasound characteristics for PCTN diagnosis

In this study, three machine learning–based quantitative models were developed to predict malignant PCTNs, derived from nine probabilistic ML models corresponding to specific ultrasound risk characteristics. These included: (1) a logistic regression model based on overall ultrasound risk characteristics, which utilized features of the entire nodule; (2) a logistic regression model based on internal ultrasound risk characteristics, focused specifically on the solid component; and (3) an integrated logistic regression model incorporating both overall and internal characteristics. Quantitative probabilities for each ultrasound risk characteristic were computed using data from 340 patients in the training cohort. Subsequently, univariate and multivariate logistic regression analyses were performed to construct the three predictive models. Model performance was evaluated through the area under the receiver operating characteristic curve (AUC), along with additional metrics including accuracy (ACC), sensitivity (SEN), specificity (SPE), positive predictive value (PPV), and negative predictive value (NPV). The DeLong test was used to compare AUCs among the three models. Finally, the predictive performance of all models was validated on an independent testing set comprising 146 PCTNs.

### Statistical analysis

Statistical analyses were conducted using R software (version 4.1.3). Categorical variables were reported as case counts, with intergroup comparisons performed using the Chi-square test or Fisher’s exact test, as appropriate. Quantitative data were first assessed for normality. Variables with a normal distribution were presented as mean ± standard deviation and compared using independent samples t-tests. Non-normally distributed data were expressed as median (interquartile range) and analyzed using the Mann-Whitney U test. A two-sided P value < 0.05 was considered indicative of statistical significance. The R code used for all analyses is provided in **Supplementary File 1**.

## Results

### Patients and ultrasound quantitative risk characteristics in PCTNs

A total of 456 patients were enrolled in the study, with a mean age of 48.69 years. The cohort included 376 females and 110 males, encompassing 486 partially cystic thyroid nodules (PCTNs) with an average size of 18.33 mm. Among these nodules, 123 (25.31%) were classified as ACR-TIRADS categories 1–3, while 363 (74.69%) were classified as categories 4–5. The training set comprised 340 PCTNs, of which 101 (29.71%) were histologically confirmed as malignant. The testing set included 146 PCTNs, with 34 cases (23.29%) identified as malignant. No statistically significant differences were observed between the training and testing cohorts with respect to clinical variables, including patient age, sex, nodule size, and anatomical distribution. Furthermore, the distribution of the nine quantitative ultrasound risk characteristics did not differ significantly between the two sets, as summarized in **Table 1**.

**Table 1:** PCTNs clinical characteristics and ultrasound risk characteristics predicted by ML probabilistic models in training and testing sets.

Characteristics	Total set (n =486)	Training set (n =340)	Testing set (n =146)	Statistic	P
Clinical characteristics					
Age (year)	48.69 ± 14.05	48.20 ± 14.01	49.83 ± 14.12	1.17	0.24
Gender, N (%)				1.42	0.23
Female	376 (77.37)	258 (75.88)	118 (80.82)		
Male	110 (22.63)	82 (24.12)	28 (19.18)		
Nodule size (mm)	18.33 ± 10.88	17.91 ± 10.38	19.31 ± 11.94	1.23	0.22
Location, n (%)				0.10	0.95
Left side	211 (43.42)	146 (42.94)	65 (44.52)		
Right side	258 (53.09)	182 (53.53)	76 (52.05)		
Isthmus	17 (3.5)	12 (3.53)	5 (3.42)		
ACR-TIRADS, n (%)				0.93	0.92
1	40 (8.23)	28 (8.24)	12 (8.22)		
2	51 (10.49)	36 (10.59)	15 (10.27)		
3	32 (6.58)	21 (6.18)	11 (7.53)		
4	223 (45.88)	160 (47.06)	63 (43.15)		
5	140 (28.81)	95 (27.94)	45 (30.82)		
Pathology result, n (%)				0.02	0.90
Benign	351 (72.22)	239 (70.29)	112 (76.71)		
Malignant	135 (27.78)	101 (29.71)	34 (23.29)		
Quantitative overall risk characteristics of entire nodule (M, (Q_1_, Q_3_))					
Taller-than-wide	0.08 (0.03, 0.21)	0.09 (0.03, 0.23)	0.07 (0.02, 0.20)	-1.22	0.22
Irregular	0.31 (0.20, 0.61)	0.33 (0.21, 0.63)	0.29 (0.19, 0.55)	-1.36	0.17
Hypoechoic	0.62 (0.35, 0.84)	0.63 (0.36, 0.85)	0.59 (0.30, 0.80)	-1.16	0.25
Microcalcifications	0.42 (0.15, 0.71)	0.45 (0.15, 0.72)	0.34 (0.13, 0.68)	-1.33	0.18
Macrocalcifications	0.16 (0.09, 0.48)	0.17 (0.10, 0.49)	0.12 (0.08, 0.41)	-1.83	0.07
Quantitative internal risk characteristics of the solid part (M, (Q_1_, Q_3_))					
Proportion≥50%	0.81 (0.39, 0.94)	0.79 (0.36, 0.93)	0.85 (0.41, 0.95)	-0.88	0.38
Eccentric configuration	0.31 (0.10, 0.67)	0.32 (0.10, 0.68)	0.31 (0.10, 0.66)	-0.33	0.74
Acute angle with cyst wall	0.08 (0.03, 0.72)	0.08 (0.03, 0.71)	0.09 (0.03, 0.79)	-0.09	0.93
Unclear margin	0.73 (0.29, 0.91)	0.71 (0.28, 0.91)	0.76 (0.32, 0.91)	-0.74	0.46

ML, Machine Learning; PCTNs, Partially Cystic Thyroid Nodules; ACR-TIRADS, The American College of Radiology Thyroid Imaging Reporting and Data System; n, number of nodules; N, number of patients; M, Median, Q_1_, 1st Quartile, Q_3_, 3st Quartile.

### Quantitative ultrasound risk characteristics between benign and malignant PCTNs

In the training set, analysis of the overall ultrasound risk characteristics of the entire nodule revealed that a taller-than-wide shape, irregular margins, hypoechogenicity, and the presence of microcalcifications were significantly associated with malignancy. In contrast, macrocalcifications did not show a statistically significant difference between benign and malignant nodules. Regarding the internal ultrasound risk characteristics of the solid component, a solid portion ≥50%, eccentric configuration, and unclear margins were significantly more frequent in malignant PCTNs, whereas the angle between the solid component and the adjacent cyst wall (acute vs. blunt) was not statistically significant. These findings are summarized in **Table 2**.

**Table 2:** In comparison of probabilities of risk characteristics predicted by ML probabilistic models between benign and malignant PCTNs in the training set.

Risk characteristics	Benign (n=239)	Malignant (n=101)	Statistic	P
Overall risk characteristics of entire nodule (M, (Q_1_, Q_3_))				
Taller-than-wide	0.07 (0.02, 0.20)	0.15 (0.05, 0.25)	2.97	2.94e-03*
Irregular	0.40 (0.23, 0.70)	0.26 (0.20, 0.42)	-3.29	9.91e-04*
Hypoechoic	0.66 (0.41, 0.87)	0.51 (0.29, 0.78)	-3.23	1.22e-03*
Microcalcification	0.33 (0.09, 0.70)	0.63 (0.39, 0.76)	-4.98	6.48e-07*
Macrocalcification	0.18 (0.09, 0.66)	0.16 (0.10, 0.32)	-1.70	8.90e-02
Internal risk characteristics of the solid part (M, (Q_1_, Q_3_))				
Proportion≥50%	0.68 (0.21, 0.92)	0.90 (0.73, 0.95)	-4.97	6.64e-07*
Eccentric configuration	0.23 (0.10, 0.61)	0.41 (0.22, 0.73)	-2.82	4.82e-03*
Acute angle with wall	0.08 (0.03, 0.88)	0.07 (0.03, 0.20)	-1.76	7.86e-02
Unclear margin	0.84 (0.54, 0.93)	0.27 (0.10, 0.49)	-9.51	1.97e-21*

ML, Machine Learning; PCTNs, Partially Cystic Thyroid Nodules; ACR-TIRADS, The American College of Radiology Thyroid Imaging Reporting and Data System; *P < 0.05, with statistical difference; M, Median, Q_1_, 1st Quartile; Q_3_, 3st Quartile.

Bold values and * both indicate statistical significance (P < 0.05).

Results from the univariate logistic regression analysis, presented in **Supplementary Table 2**, identified the following as independent risk factors for predicting malignancy in PCTNs: irregular margins, hypoechogenicity, microcalcifications, macrocalcifications, solid component proportion ≥50%, eccentric configuration, an acute angle between the solid part and the adjacent cyst wall, and unclear internal margins.

### Three ML quantitative models in predicting malignant PCTNs

The results of the multivariate logistic regression analysis are presented in **Supplementary Table 3**. Among the overall ultrasound risk characteristics of the entire nodule, hypoechogenicity, microcalcifications, and macrocalcifications were identified as statistically significant predictors. From the internal ultrasound characteristics of the solid component, a solid portion ≥50% and unclear margins also demonstrated significant associations with malignancy.

**Figure 3:**
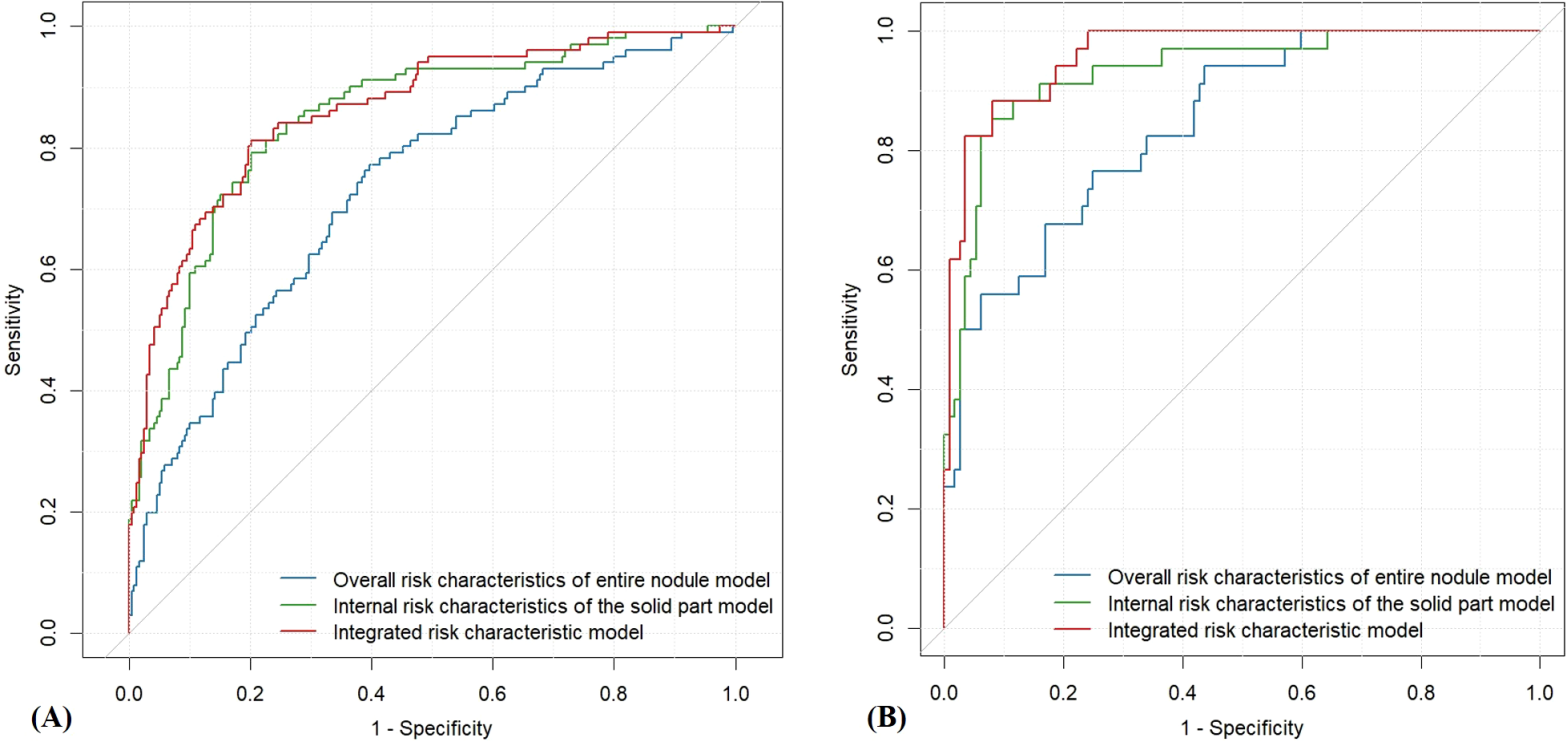
ROC curves of the three quantitative ultrasound risk characteristics models. **(A)** Training Set; **(B)** Testing Set.

In this study, three machine learning–based quantitative models were developed to predict malignant PCTNs, utilizing probabilities generated from ML models of individual ultrasound risk characteristics. Among these, the integrated ML model—which incorporated both overall and internal ultrasound characteristics—demonstrated the highest performance. In the training set, it achieved an AUC of 0.86 (0.82-0.91) and an accuracy of 0.80 (0.76-0.84). In the testing set, the integrated model significantly outperformed the model based solely on overall characteristics, with an AUC of 0.96 (0.93-0.99) versus 0.85 (0.77-0.92) (P = 2.35e^-6^) and an accuracy of 0.91(0.85-0.95) compared to 0.75 (0.68-0.82). However, no statistically significant difference was observed when compared to the internal characteristics–based model (AUC: 0.96 (0.93-0.99) vs. 0.93 (0.88-0.98), P =1.01e^-1^). Detailed performance metrics of the three models are provided in **Table 3** and illustrated in **Figure 3**.

**Table 3:** In comparison of the performance of three ML quantitative models in predicting malignant PCTNs in the training and testing sets.

ML Model	Training set (n=340)			Testing set (n=146)		
	1	2	3	1	2	3
SEN (%) [95% CI]	0.77 (0.68, 0.85)	0.79 (0.70-0.87)	0.81 (0.72-0.88)	0.76 (0.59-0.89)	0.85 (0.69-0.95)	0.88 (0.73-0.97)
SPE (%) [95% CI]	0.60 (0.54-0.67)	0.80 (0.74-0.85)	0.80 (0.74-0.85)	0.75 (0.66-0.83)	0.92 (0.85-0.96)	0.92 (0.85-0.96)
ACC (%) [95% CI]	0.65 (0.60-0.70)	0.80 (0.75-0.84)	0.80 (0.76-0.84)	0.75 (0.68-0.82)	0.90 (0.84-0.95)	0.91 (0.85-0.95)
NPV (%) [95% CI]	0.86 (0.80-0.91)	0.90 (0.85-0.94)	0.91 (0.86-0.94)	0.91 (0.84-0.96)	0.95 (0.90-0.98)	0.96 (0.91-0.99)
PPV (%) [95% CI]	0.45 (0.38-0.53)	0.62 (0.54-0.71)	0.63 (0.54-0.71)	0.48 (0.34-0.62)	0.76 (0.60-0.89)	0.77 (0.61-0.89)
AUROC [95% CI]	0.73 (0.67-0.79)	0.85 (0.80-0.89)	0.86 (0.82-0.91)	0.85 (0.77-0.92)	0.93 (0.88-0.98)	0.96 (0.93-0.99)
Cutoff value	0.25	0.30	0.31	0.32	0.48	0.41

ML, Machine Learning; ML Model 1, Overall risk characteristics of entire nodule model; ML Model 2, Internal risk characteristics of the solid part model; ML Model 3, Integrated risk characteristic model; PCTNs, Partially Cystic Thyroid Nodules.

## Discussion

Partially cystic thyroid nodules (PCTNs) represent a distinct subset of thyroid nodules characterized by the coexistence of cystic and solid components. These nodules are typically considered benign and are frequently assigned to ACR-TIRADS categories 2 or 3. However, a considerable proportion of atypical or suspicious PCTNs are classified as categories 4 or 5, prompting clinical recommendations for further evaluation via fine-needle aspiration (FNA) or surgical intervention. This clinical variability raises critical questions: which ultrasound characteristics are most indicative of malignancy, and how can malignant PCTNs be identified with greater accuracy? These challenges have garnered growing interest in recent research. Nevertheless, most existing studies rely heavily on the subjective interpretation of ultrasound characteristics by clinicians, introducing variability and potential diagnostic inconsistency.

In the present study, machine learning techniques were employed to quantitatively assess both the overall and internal ultrasound characteristics of PCTNs. Based on these assessments, three interpretable quantitative models were constructed to facilitate the differentiation between benign and malignant nodules. These models not only estimate the likelihood of malignancy independently of physician experience but also generate textual outputs that are clinically interpretable and supportive of diagnostic decision-making.

A total of 486 nodules were included in the analysis, among which 135 (27.78%) were pathologically confirmed as malignant—a rate notably higher than the previously reported average malignancy rate of 17.6% for PCTNs. This elevated proportion may be attributed to our institution’s role as a specialized referral center for thyroid nodule diagnosis and management, which receives a substantial number of complex cases from regional hospitals.

Among the five overall ultrasound risk characteristics of nodules identified by ML probabilistic models, hypoechogenicity, microcalcifications and macrocalcifications were significantly associated with malignant PCTNs. Specifically, PCTNs with microcalcifications exhibited a risk of malignancy 7.62 times higher. Similar findings have been reported by other researchers (6, 15), however, the independent diagnostic accuracies of these risk characteristics in predicting malignant PCTNs are limited, approximately ranging from 0.5 to 0.8 (5, 10). In this study, macrocalcifications did not show a statistically significant difference in simple group comparisons, but both univariate and multivariate logistic regression identified them as predictors of malignancy. This discrepancy likely reflects that logistic regression models capture the strength of association with malignancy risk, rather than just differences in occurrence rates between benign and malignant groups. Then we proposed ML quantitative models that integrate these overall ultrasound risk characteristics, achieving an accuracy of 0.75, with a specificity of 0.75 and a sensitivity of 0.76 in the testing set. By objectively quantifying ultrasound risk characteristics through ML, we can reduce subjective bias in physician judgment, thereby improving the diagnostic accuracy of PCTNs. This advantage is particularly valuable for junior doctors with less experience.

Our study found that for the solid part of PCTNs, when the proportion of the solid part is ≥50% and unclear margins are more likely to indicate malignancy. Among these factors, PCTNs with a solid part proportion ≥50% exhibit a significantly higher risk, with an 11.65 times greater likelihood of malignancy. These characteristics are closely related to the inherent biological behavior of tumor tissue. Other research teams have also suggested that the acute angle between the solid component and the adjacent cyst wall is associated with malignant PCTNs (16). This acute angle may result from the true tumor tissue in malignant PCTNs originating from pedunculated lesions on the wall of a thyroid cyst and then growing toward the center of the nodule (17, 18). However, our study did not demonstrate a significant association in this regard. This discrepancy may be attributable to the retrospective nature of the study, as determining the angle between the solid component and the cyst wall may require dynamic, real-time ultrasound scanning rather than reliance on a single static image. Future prospective studies incorporating real-time imaging protocols may be better suited to evaluate this characteristic accurately. Notably, the diagnostic performance of the ML-based quantitative model utilizing the significant internal ultrasound risk characteristics yielded promising results, with an accuracy of 0.90, specificity of 0.92, and sensitivity of 0.85.

**Figure 4:**
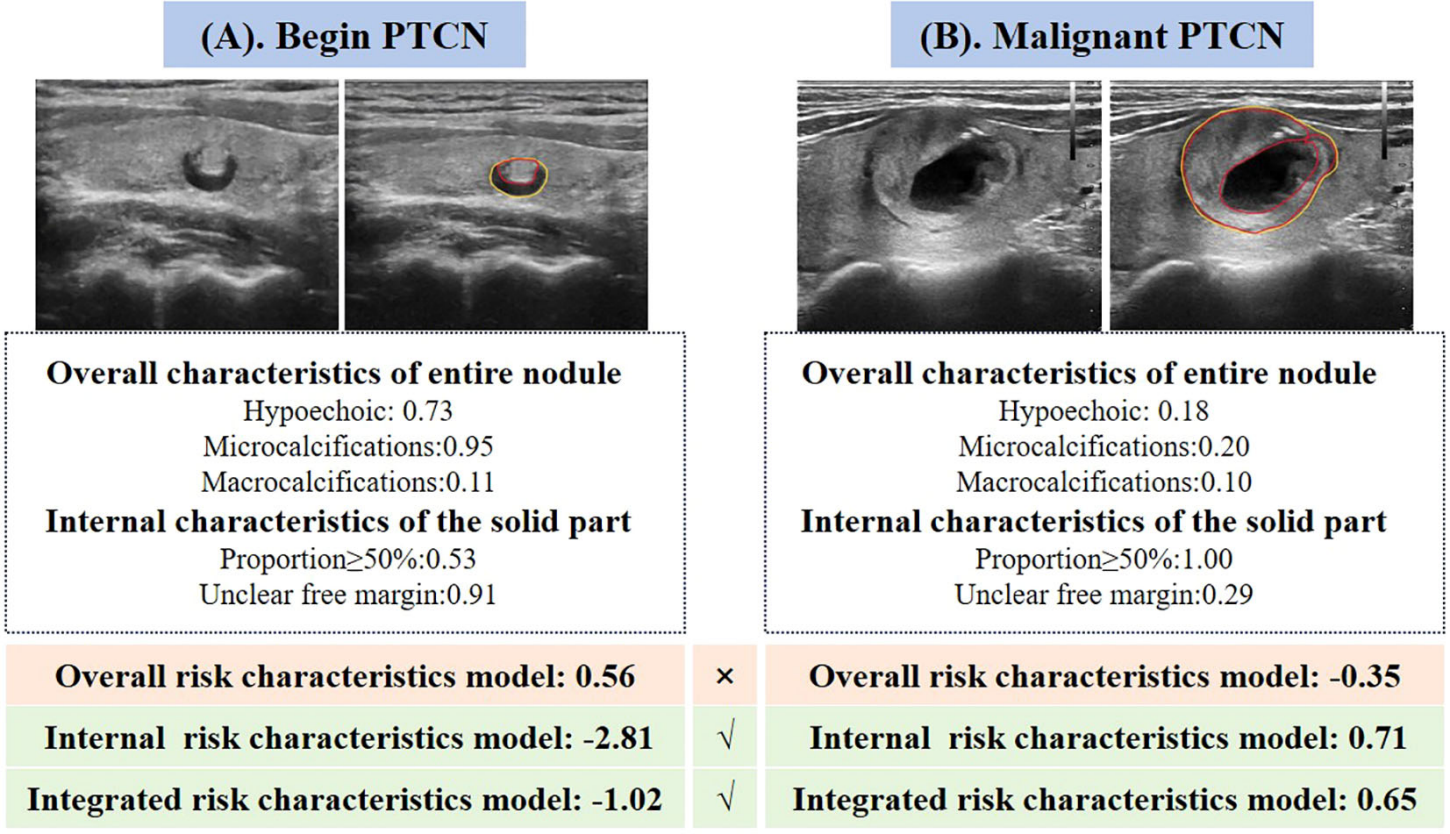
Two representative cases illustrating the performance of the three quantitative ultrasound risk characteristics models in predicting benign and malignant PCTNs. **(A)** A 70-year-old female patient with a pathologically confirmed benign nodule was incorrectly classified as malignant by the model based on overall ultrasound risk characteristics. In contrast, both the internal risk characteristics model and the integrated model accurately identified the nodule as benign. **(B)** A 31-year-old female patient with a pathologically confirmed malignant nodule was misclassified as benign by the overall risk characteristics model. However, the internal and integrated models both correctly predicted the malignancy.

Among the three predictive models, the integrated model demonstrated the highest diagnostic performance (AUC = 0.96), followed by the model based on internal ultrasound characteristics (AUC = 0.93), with no statistically significant difference between the two. Both models significantly outperformed the model relying solely on overall nodule characteristics (AUC = 0.85). These findings suggest that accurate identification of internal ultrasound characteristics plays a more pivotal role in determining the malignancy risk of PCTNs. **Figure 4** illustrates two clinical cases of PCTNs, in which models that accounted for internal ultrasound characteristics achieved accurate differentiation between benign and malignant PCTNs. Accordingly, clinicians are encouraged to place greater emphasis on evaluating the morphology and location of the solid component, as well as its spatial relationship with the surrounding cyst wall during ultrasound assessment.

In the testing cohort of 146 PCTNs, the misdiagnosis rates for the overall risk characteristics model, internal characteristics model, and integrated model were 13.70% (20 nodules), 12.33% (18 nodules), and 8.22% (12 nodules), respectively. All three models achieved high performance in identifying benign nodules, as reflected by their low false positive rates (FPRs) ranging from 2.68% to 5.36%. However, the false negative rates (FNRs) for malignant nodules varied more markedly, at 50.00%, 35.29%, and 17.65%, respectively, highlighting the superior capability of the integrated model in correctly diagnosing malignant PCTNs. Representative examples are provided in **Figure 4**.

Despite the demonstrated utility of ML-based quantitative models in this study, several limitations must be acknowledged. First, this was a retrospective, single-center study, which may introduce selection bias. Additionally, the annotation of ultrasound risk characteristics was manually performed by experienced radiologists, which, despite their expertise, may introduce subjectivity. Future studies should incorporate quantitative inter-reader agreement analysis to assess annotation consistency. Moreover, the current model functions as an independent decision support tool on the Darwin scientific research platform, requiring manual delineation of ultrasound images for feature extraction; integration into clinical information systems remains a goal for future work. Future prospective, multi-center studies are warranted to externally validate the model’s performance across diverse populations and imaging systems. Nonetheless, the proposed approach for quantifying ultrasound characteristics via machine learning offers significant potential for application in other disease types and across various medical imaging modalities.

## Conclusions

The internal ultrasound characteristics of the solid component within PCTNs play a critical role in differentiating between benign and malignant lesions. Machine learning models that integrate both overall and internal nodule characteristics not only enhance the diagnostic accuracy for PCTNs, but also generate interpretable outputs that can be readily understood and applied by clinicians in routine practice.

## Data Availability

The original contributions presented in the study are included in the article/**Supplementary Material**. Further inquiries can be directed to the corresponding author.
