# Supplementary material for: Machine learning–based quantification of overall and internal ultrasound characteristics for diagnosing malignant partially cystic thyroid nodules
Source: Front Endocrinol (Lausanne). 2025 Aug 6;16:1635122. doi: 10.3389/fendo.2025.1635122 (PMC12364630; doi:10.3389/fendo.2025.1635122)
Supplement: Supplementary file 3 [file Table1.docx]

S Table 1 Risk prediction score formulas for nine quantitative ultrasound risk characteristics.

| Risk characteristics | Initial feature number | Filtered features number | Risk score formula |
| --- | --- | --- | --- |
| Taller-than-wide | 1125 | 10 | +1.120*original_shape2D_Elongation_LR_paramsName1+0.824*square_glcm_InverseVariance_LR_paramsName1+0.364*wavelet-HL_firstorder_Skewness_LR_paramsName1+0.352*exponential_glszm_GrayLevelNonUniformityNormalized_LR_paramsName1-0.349*lbp-2D_firstorder_Entropy_LR_paramsName1-0.349*lbp-2D_glcm_DifferenceEntropy_LR_paramsName1-0.349*lbp-2D_glcm_JointEntropy_LR_paramsName1-0.349*lbp-2D_glcm_SumEntropy_LR_paramsName1+0.344*wavelet-LH_firstorder_Skewness_LR_paramsName1+0.315*logarithm_firstorder_Minimum_LR_paramsName1-1.658 |
| Irregular | 1125 | 17 | +0.746*log-sigma-3-0-mm-3D_firstorder_Mean_LR_paramsName1-0.574*wavelet-HL_firstorder_Median_LR_paramsName1-0.451*square_glcm_InverseVariance_LR_paramsName1+0.311*gradient_firstorder_Kurtosis_LR_paramsName1-0.183*wavelet-HH_firstorder_Mean_LR_paramsName1-0.171*lbp2D_glszm_ZoneVariance_LR_paramsName1+0.171*exponential_ngtdm_Coarseness_LR_paramsName1-0.146*original_shape2D_Sphericity_LR_paramsName1-0.127*wavelet-HL_glszm_ZoneEntropy_LR_paramsName1-0.120*wavelet-HL_firstorder_Skewness_LR_paramsName1-0.111*wavelet-LH_firstorder_Skewness_LR_paramsName1+0.092*exponential_firstorder_Kurtosis_LR_paramsName1-0.086*gradient_ngtdm_Busyness_LR_paramsName1-0.050*lbp-2D_firstorder_Entropy_LR_paramsName1-0.050*lbp-2D_glcm_DifferenceEntropy_LR_paramsName1-0.050*lbp-2D_glcm_JointEntropy_LR_paramsName1-0.050*lbp-2D_glcm_SumEntropy_LR_paramsName1-0.343 |
| Hypoechoic | 1125 | 10 | +0.862*square_firstorder_InterquartileRange_LR_paramsName1+0.375*square_glrlm_RunLengthNonUniformityNormalized_LR_paramsName1+0.330*square_glcm_SumAverage_LR_paramsName1+0.330*square_glcm_JointAverage_LR_paramsName1-0.317*square_glcm_SumEntropy_LR_paramsName1-0.208*square_firstorder_Entropy_LR_paramsName1+0.177*square_glcm_JointEntropy_LR_paramsName1-0.082*square_glcm_Idm_LR_paramsName1-0.029*square_firstorder_MeanAbsoluteDeviation_LR_paramsName1+0.140 |
| Microcalcifications | 1125 | 10 | +0.944*squareroot_firstorder_Skewness_LR_paramsName1+0.690*log-sigma-3-0-mm-3D_firstorder_Median_LR_paramsName1-0.681*lbp-2D_glrlm_RunLengthNonUniformityNormalized_LR_paramsName1-0.490*original_firstorder_Skewness_LR_paramsName1+0.175*log-sigma-3-0-mm-3D_glcm_Idn_LR_paramsName1-0.165*log-sigma-3-0-mm-3D_firstorder_Skewness_LR_paramsName1+0.121*log-sigma-3-0-mm-3D_glcm_Idmn_LR_paramsName1-0.099*wavelet-LL_firstorder_Skewness_LR_paramsName1-0.827 |
| Macrocalcifications | 1125 | 15 | +0.421*wavelet-LL_firstorder_Skewness_LR_paramsName1-0.409*square_ngtdm_Busyness_LR_paramsName1+0.400*exponential_glcm_ClusterShade_LR_paramsName1+0.332*lbp-2D_firstorder_InterquartileRange_LR_paramsName1+0.276*exponential_glrlm_GrayLevelVariance_LR_paramsName1+0.266*wavelet-LH_firstorder_Mean_LR_paramsName1-0.166*lbp-2D_firstorder_Entropy_LR_paramsName1-0.166*lbp-2D_glcm_DifferenceEntropy_LR_paramsName1-0.166*lbp-2D_glcm_JointEntropy_LR_paramsName1-0.166*lbp-2D_glcm_SumEntropy_LR_paramsName1-0.166*lbp-2D_glszm_ZoneEntropy_LR_paramsName1+0.158*wavelet-LL_firstorder_Kurtosis_LR_paramsName1-0.108*gradient_firstorder_Minimum_LR_paramsName1-0.095*lbp-2D_glrlm_RunLengthNonUniformity_LR_paramsName1-1.053 |
| Eccentric configuration | 1125 | 22 | +1.295*lbp-2D_gldm_DependenceEntropy_LR_paramsName1+0.825*logarithm_glrlm_RunEntropy_LR_paramsName1-0.576*wavelet-HH_glcm_MCC_LR_paramsName1-0.489*log-sigma-3-0-mm-3D_firstorder_Skewness_LR_paramsName1-0.470*wavelet-LH_firstorder_Median_LR_paramsName1-0.412*logarithm_firstorder_Minimum_LR_paramsName1-0.401*wavelet-HL_glcm_Imc1_LR_paramsName1-0.313*wavelet-LH_firstorder_Mean_LR_paramsName1-0.272*wavelet-HH_glszm_SmallAreaEmphasis_LR_paramsName1+0.256*wavelet-LH_glcm_MCC_LR_paramsName1-0.249*wavelet-HL_firstorder_Median_LR_paramsName1-0.212*wavelet-LH_glcm_Correlation_LR_paramsName1-0.202*log-sigma-3-0-mm-3D_glcm_ClusterShade_LR_paramsName1-0.180*square_ngtdm_Coarseness_LR_paramsName1-0.160*wavelet-LH_glszm_LargeAreaHighGrayLevelEmphasis_LR_paramsName1-0.149*wavelet-LH_gldm_DependenceNonUniformityNormalized_LR_paramsName1+0.147*wavelet-HL_glszm_LargeAreaHighGrayLevelEmphasis_LR_paramsName1+0.138*wavelet-HL_glszm_GrayLevelVariance_LR_paramsName1+0.110*wavelet-HL_firstorder_Skewness_LR_paramsName1-0.058*wavelet-HH_firstorder_Mean_LR_paramsName1+0.031*wavelet-LL_glrlm_LongRunLowGrayLevelEmphasis_LR_paramsName1-0.019*original_shape2D_Sphericity_LR_paramsName1+0.125 |
| Acute angle with cyst wall | 1125 | 21 | +0.889*original_shape2D_Sphericity_LR_paramsName1-0.883*wavelet-HL_glszm_GrayLevelVariance_LR_paramsName1-0.550*wavelet-HL_glcm_Correlation_LR_paramsName1+0.530*squareroot_firstorder_Kurtosis_LR_paramsName1+0.514*log-sigma-3-0-mm-3D_firstorder_Mean_LR_paramsName1-0.430*gradient_glrlm_ShortRunLowGrayLevelEmphasis_LR_paramsName1-0.409*wavelet-LH_firstorder_Kurtosis_LR_paramsName1+0.396*wavelet-LH_firstorder_Energy_LR_paramsName1+0.396*wavelet-LH_firstorder_TotalEnergy_LR_paramsName1+0.322*original_shape2D_Elongation_LR_paramsName1+0.227*logarithm_firstorder_Kurtosis_LR_paramsName1+0.169*lbp-2D_firstorder_Entropy_LR_paramsName1+0.169*lbp-2D_glcm_DifferenceEntropy_LR_paramsName1+0.169*lbp-2D_glcm_JointEntropy_LR_paramsName1+0.169*lbp-2D_glcm_SumEntropy_LR_paramsName1+0.169*lbp-2D_glszm_ZoneEntropy_LR_paramsName1+0.038*wavelet-LH_firstorder_Median_LR_paramsName1-0.032*lbp-2D_glrlm_ShortRunEmphasis_LR_paramsName1-0.032*lbp-2D_glrlm_ShortRunHighGrayLevelEmphasis_LR_paramsName1-0.032*lbp-2D_glrlm_ShortRunLowGrayLevelEmphasis_LR_paramsName1+0.634 |
| Proportion≥50% | 1125 | 12 | +1.086*square_glcm_InverseVariance_LR_paramsName1+0.938*wavelet-LH_glrlm_RunEntropy_LR_paramsName1+0.644*wavelet-HH_firstorder_Mean_LR_paramsName1-0.628*gradient_glcm_MCC_LR_paramsName1-0.578*squareroot_firstorder_Skewness_LR_paramsName1-0.321*squareroot_glcm_ClusterShade_LR_paramsName1+0.131*lbp-2D_firstorder_Entropy_LR_paramsName1+0.131*lbp-2D_glcm_DifferenceEntropy_LR_paramsName1+0.131*lbp-2D_glcm_JointEntropy_LR_paramsName1+0.131*lbp-2D_glcm_SumEntropy_LR_paramsName1-0.129*wavelet-LL_glrlm_LowGrayLevelRunEmphasis_LR_paramsName1+0.934 |
| Unclear margin | 1125 | 21 | -0.773*wavelet-HL_firstorder_Mean_LR_paramsName1+0.750*wavelet-HH_glcm_InverseVariance_LR_paramsName1+0.681*square_glcm_JointAverage_LR_paramsName1+0.620*wavelet-LH_ngtdm_Contrast_LR_paramsName1-0.611*logarithm_firstorder_Minimum_LR_paramsName1-0.534*log-sigma-3-0-mm-3D_firstorder_Mean_LR_paramsName1+0.459*lbp-2D_glrlm_RunLengthNonUniformityNormalized_LR_paramsName1+0.433*lbp-2D_firstorder_InterquartileRange_LR_paramsName1+0.417*wavelet-HH_firstorder_InterquartileRange_LR_paramsName1-0.330*wavelet-LH_glrlm_RunEntropy_LR_paramsName1+0.302*wavelet-HL_glcm_ClusterShade_LR_paramsName1-0.299*squareroot_firstorder_Skewness_LR_paramsName1+0.274*wavelet-HL_glcm_Imc1_LR_paramsName1+0.214*wavelet-HL_glszm_LargeAreaHighGrayLevelEmphasis_LR_paramsName1-0.184*original_shape2D_Elongation_LR_paramsName1+0.161*exponential_glszm_SizeZoneNonUniformity_LR_paramsName1-0.147*wavelet-LH_glcm_Idmn_LR_paramsName1+0.130*wavelet-HL_firstorder_Kurtosis_LR_paramsName1+0.067*logarithm_glszm_LargeAreaHighGrayLevelEmphasis_LR_paramsName1+0.033*wavelet-HL_glrlm_RunVariance_LR_paramsName1-0.007*wavelet-LH_glcm_Correlation_LR_paramsName1+0.251 |

S Table 2 The univariate logistic analysis of the PCTNs risk characteristics quantified by machine learning in training set.

|  | B | SE | P value | OR (95%CI) |
| --- | --- | --- | --- | --- |
| **Quantitative overall risk characteristics of entire nodule** | | | | |
| Taller-than-wide | 0.66 | 0.65 | 3.05e-01 | 1.94(0.54, 6.93) |
| Irregular | 1.53 | 0.46 | **8.08e-04*** | 0.22(0.09, 0.53) |
| Hypoechoic | -1.47 | 0.45 | **1.15e-03*** | 0.23(0.09, 0.56) |
| Microcalcifications | 2.03 | 0.42 | **1.24e-06*** | 7.62(3.35, 17.31) |
| Macrocalcifications | -1.38 | 0.46 | **3.01e-03*** | 0.25(0.10, 0.63) |
| **Quantitative internal risk characteristics of the solid part** | | | | |
| Proportion≥50% | 2.46 | 0.47 | **1.41e-07*** | 11.65(4.67, 29.08) |
| Eccentric configuration | 0.96 | 0.38 | **1.04e-02*** | 2.62(1.25, 5.48) |
| Acute angle with cyst wall | -1.69 | 0.41 | **2.92e-05*** | 0.18(0.08, 0.41) |
| Unclear margin | 4.00 | 0.46 | **6.78e-18*** | 0.02(0.01, 0.05) |

PCTNs: Partially Cystic Thyroid Nodules; B: Regression coefficient; SE: standard error; OR: Odd Radio; CI: Confidence Interval.; *: P < 0.05, with statistical difference.

S Table 3 Three models of machine learning based on multivariate logistic regression to predict malignant PCTNs categorized TIRADS 4 and 5 in training set

|  | B | SE | P | OR (95%CI) |
| --- | --- | --- | --- | --- |
| **ML Model 1: Overall risk characteristics of entire nodule** | | | | |
| Constant | -0.42 | 0.40 | 2.96e-01 | 0.66(0.30, 1.45) |
| Hypoechoic | -1.34 | 0.51 | **8.84e-03*** | 0.26(0.09, 0.71) |
| Irregular | -0.97 | 0.51 | 5.77e-02 | 0.38(0.14, 1.01) |
| Microcalcifications | 2.21 | 0.45 | **9.18e-07*** | 9.16(3.85, 22.67) |
| Macrocalcifications | -1.24 | 0.55 | **2.44e-02*** | 0.29(0.09, 0.83) |
| **ML Model 2: Internal risk characteristics of the solid part** | | | | |
| Constant | -0.41 | 0.52 | 4.29e-01 | 0.66(0.23, 1.80) |
| Proportion≥50% | 2.32 | 0.53 | **1.16e-05*** | 10.23(3.79, 30.59) |
| Acute angle with cyst wall | 0.16 | 0.58 | 7.85e-01 | 1.17(0.37, 3.67) |
| Unclear margin | -4.01 | 0.57 | **1.79e-12*** | 0.02(0.01, 0.05) |
| Eccentric configuration | -0.04 | 0.50 | 9.29e-01 | 0.96(0.36, 2.54) |
| **ML Model 3: Integrated the overall and** **internal risk characteristics** | | | | |
| Constant | -1.03 | 0.78 | 1.86e-01 | 0.36(0.07, 1.62 |
| Hypoechoic | -0.86 | 0.62 | 1.67e-01 | 0.42(0.12, 1.42) |
| Irregular | -0.26 | 0.63 | 6.80e-01 | 0.77(0.22, 2.68) |
| Microcalcifications | 2.03 | 0.58 | **5.10e-04*** | 7.61(2.46,24.52) |
| Macrocalcifications | 0.21 | 0.79 | 7.91e-01 | 1.23(0.26, 5.88) |
| Proportion≥50% | 2.32 | 0.54 | **2.11e-05*** | 10.13(3.65, 31.18) |
| Acute angle with cyst wall | -0.31 | 0.77 | 6.88e-01 | 0.73(0.16, 3.29) |
| Unclear margin | -3.48 | 0.61 | **9.89e-09*** | 0.03(0.01, 0.10) |
| Eccentric configuration | 0.08 | 0.52 | 8.71e-01 | 1.09(0.39, 3.02) |

ML: Machine Learning; PCTNs: Partially Cystic Thyroid Nodules; B: Regression coefficient; OR: Odd Radio; CI: Confidence Interval; *: P < 0.05, with statistical difference.


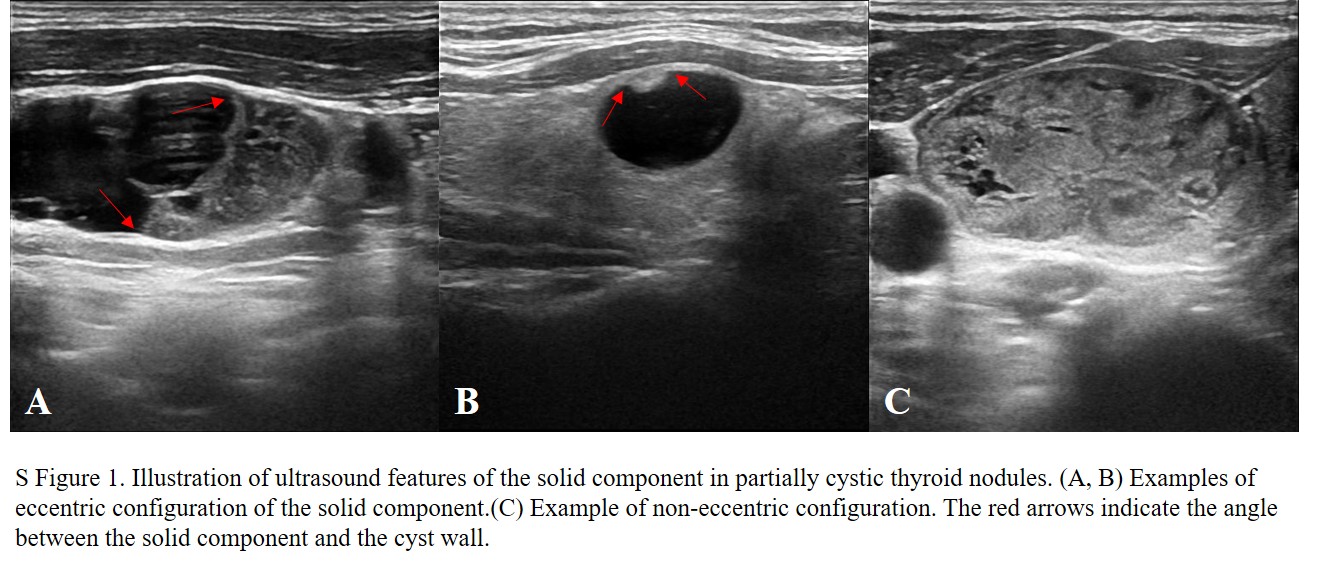


Supplement File 1----R Code

yutong zhang

2025年7月8日

Table of Contents

**未找到目录项。**

# 加载必要的包
library(xlsx)

# 设置文件路径并读取数据
training <- "E:/R/PCTN/486 ML model/Training data.xlsx"
train_data <- read.xlsx(training, 1)
head(train_data)

## pathology Microcalcifications Hypoechoic Macrocalcifications
## 1 1 0.6167923 0.7494476 0.14990073
## 2 1 0.6280004 0.8736674 0.11617793
## 3 1 0.2400723 0.2967649 0.09708326
## 4 0 0.3231283 0.3827187 0.03646531
## 5 1 0.4518859 0.6640778 0.18619312
## 6 1 0.8170030 0.1563390 0.53302995
## Unclear.free.margin Eccentric.growth Acute.angle.with.cyst.wall
## 1 0.24786455 0.9616722 0.07424668
## 2 0.81833381 0.9569555 0.08176256
## 3 0.45088426 0.3018699 0.34498470
## 4 0.23121483 0.7704886 0.03868033
## 5 0.64720025 0.6504896 0.10779055
## 6 0.02408844 0.9364066 0.11127165
## Proportion.50. Taller Lobulated.or.irregular
## 1 0.5328585 0.06390751 0.4214795
## 2 0.1019633 0.10817741 0.4622934
## 3 0.6010154 0.41473679 0.4521006
## 4 0.8122411 0.25938025 0.2214161
## 5 0.9231735 0.05841650 0.1740907
## 6 0.9713495 0.33075131 0.2046442

View(train_data)


train_data$Unclear.free.margin<--train_data$Unclear.free.margin
train_data$Lobulated.or.irregular<--train_data$Lobulated.or.irregular

###univariate logistic regression
uni_logistic <- lapply(train_data[,-1], function(x) {
 fit <- glm(pathology ~ x, family = binomial, data = train_data)
 summary_fit <- summary(fit)

 coef <- summary_fit$coef
 OR <- exp(coef[2, "Estimate"]) # 计算OR值，取第二行的Estimate对应于x的回归系数
 lower_CI <- exp(coef[2, "Estimate"] - 1.96 * coef[2, "Std. Error"]) # 95%CI下限
 upper_CI <- exp(coef[2, "Estimate"] + 1.96 * coef[2, "Std. Error"]) # 95%CI上限

 # 将OR和95%置信区间加入到summary_fit的系数结果中
 summary_fit$coef <- cbind(summary_fit$coef, OR = OR, `95% CI Lower` = lower_CI, `95% CI Upper` = upper_CI)

 return(summary_fit)
})

# 提取每个单变量的回归结果并命名
univariate_test_results <- list()
for (i in 1:length(uni_logistic)) {
 univariate_test_results[[i]] <- uni_logistic[[i]]$coef
 names(univariate_test_results)[[i]] <- names(uni_logistic)[[i]]
}
univariate_test_results

## $Microcalcifications
## Estimate Std. Error z value Pr(>|z|) OR 95% CI Lower
## (Intercept) -1.844662 0.2506586 -7.359261 1.849316e-13 7.61803 3.352657
## x 2.030518 0.4187575 4.848911 1.241410e-06 7.61803 3.352657
## 95% CI Upper
## (Intercept) 17.30997
## x 17.30997
##
## $Hypoechoic
## Estimate Std. Error z value Pr(>|z|) OR
## (Intercept) -0.007277855 0.2808137 -0.02591702 0.979323523 0.2290433
## x -1.473844398 0.4534615 -3.25020870 0.001153203 0.2290433
## 95% CI Lower 95% CI Upper
## (Intercept) 0.09417233 0.5570725
## x 0.09417233 0.5570725
##
## $Macrocalcifications
## Estimate Std. Error z value Pr(>|z|) OR 95% CI Lower
## (Intercept) -0.4604435 0.172286 -2.672552 0.007527660 0.2517395 0.1011918
## x -1.3793606 0.464988 -2.966443 0.003012659 0.2517395 0.1011918
## 95% CI Upper
## (Intercept) 0.6262635
## x 0.6262635
##
## $Unclear.free.margin
## Estimate Std. Error z value Pr(>|z|) OR 95% CI Lower
## (Intercept) 1.248169 0.2565204 4.865769 1.140124e-06 54.72626 22.02435
## x 4.002344 0.4643853 8.618584 6.778694e-18 54.72626 22.02435
## 95% CI Upper
## (Intercept) 135.9842
## x 135.9842
##
## $Eccentric.growth
## Estimate Std. Error z value Pr(>|z|) OR 95% CI Lower
## (Intercept) -1.2632236 0.2027456 -6.230585 4.646973e-10 2.622486 1.254059
## x 0.9641227 0.3763966 2.561454 1.042350e-02 2.622486 1.254059
## 95% CI Upper
## (Intercept) 5.484139
## x 5.484139
##
## $Acute.angle.with.cyst.wall
## Estimate Std. Error z value Pr(>|z|) OR 95% CI Lower
## (Intercept) -0.4404562 0.1456316 -3.024455 2.490817e-03 0.1839111 0.0831338
## x -1.6933026 0.4051027 -4.179934 2.915937e-05 0.1839111 0.0831338
## 95% CI Upper
## (Intercept) 0.4068538
## x 0.4068538
##
## $Proportion.50.
## Estimate Std. Error z value Pr(>|z|) OR 95% CI Lower
## (Intercept) -2.590717 0.3776128 -6.860778 6.848672e-12 11.65313 4.670131
## x 2.455574 0.4665242 5.263552 1.412984e-07 11.65313 4.670131
## 95% CI Upper
## (Intercept) 29.07742
## x 29.07742
##
## $Taller
## Estimate Std. Error z value Pr(>|z|) OR 95% CI Lower
## (Intercept) -0.9688369 0.1601192 -6.050723 1.441968e-09 1.944257 0.54553
## x 0.6648801 0.6484069 1.025406 3.051718e-01 1.944257 0.54553
## 95% CI Upper
## (Intercept) 6.929292
## x 6.929292
##
## $Lobulated.or.irregular
## Estimate Std. Error z value Pr(>|z|) OR 95% CI Lower
## (Intercept) -0.2534889 0.2079212 -1.219159 0.2227838879 4.622914 1.887471
## x 1.5310253 0.4570344 3.349912 0.0008083717 4.622914 1.887471
## 95% CI Upper
## (Intercept) 11.32274
## x 11.32274

###multivariate logistic regression, significant factors only are analyzed

# Quantitative overall risk characteristics of entire nodule-------ACR_TIRADS_characteristics=overall risk characteristics
ACR_TIRADS_Chara <- glm(pathology ~ Microcalcifications + Macrocalcifications + Lobulated.or.irregular + Hypoechoic, family = binomial, data = train_data)
summary_ACR <- summary(ACR_TIRADS_Chara)
ACR_OR <- exp(coef(ACR_TIRADS_Chara))
ACR_CI <- exp(confint(ACR_TIRADS_Chara))
ACR_result <- cbind(summary_ACR$coef, OR = ACR_OR, `95% CI Lower` = ACR_CI[, 1], `95% CI Upper` = ACR_CI[, 2])
ACR_result

## Estimate Std. Error z value Pr(>|z|) OR
## (Intercept) -0.4214368 0.4035434 -1.044341 2.963278e-01 0.6561035
## Microcalcifications 2.2145700 0.4511662 4.908545 9.175436e-07 9.1574706
## Macrocalcifications -1.2444885 0.5528330 -2.251111 2.437849e-02 0.2880882
## Lobulated.or.irregular 0.9679509 0.5099946 1.897963 5.770095e-02 2.6325445
## Hypoechoic -1.3395676 0.5116395 -2.618186 8.839856e-03 0.2619589
## 95% CI Lower 95% CI Upper
## (Intercept) 0.29592271 1.4466246
## Microcalcifications 3.84852585 22.6664013
## Macrocalcifications 0.09374773 0.8263942
## Lobulated.or.irregular 0.98640892 7.3521578
## Hypoechoic 0.09467388 0.7070826

# Quantitative internal risk characteristics of the solid part---------Extra_characteristics=internal risk characteristics
Extra_Chara <- glm(pathology ~ Unclear.free.margin + Acute.angle.with.cyst.wall + Proportion.50.+Eccentric.growth, family = binomial, data = train_data)
summary_Extra <- summary(Extra_Chara)
Extra_OR <- exp(coef(Extra_Chara))
Extra_CI <- exp(confint(Extra_Chara))
Extra_result <- cbind(summary_Extra$coef, OR = Extra_OR, `95% CI Lower` = Extra_CI[, 1], `95% CI Upper` = Extra_CI[, 2])
Extra_result

## Estimate Std. Error z value Pr(>|z|)
## (Intercept) -0.41001223 0.5180762 -0.79141297 4.287030e-01
## Unclear.free.margin 4.01277899 0.5691930 7.04994382 1.789900e-12
## Acute.angle.with.cyst.wall 0.15916642 0.5829761 0.27302391 7.848348e-01
## Proportion.50. 2.32489328 0.5302179 4.38478841 1.160986e-05
## Eccentric.growth -0.04428669 0.4982893 -0.08887747 9.291793e-01
## OR 95% CI Lower 95% CI Upper
## (Intercept) 0.6636421 0.2324713 1.795670
## Unclear.free.margin 55.3003360 18.9933237 178.309390
## Acute.angle.with.cyst.wall 1.1725331 0.3679227 3.674882
## Proportion.50. 10.2255888 3.7855246 30.585550
## Eccentric.growth 0.9566796 0.3574529 2.536217

# Integrated the overall and internal risk characteristics
integrate_Chara <- glm(pathology ~ Microcalcifications + Macrocalcifications + Hypoechoic + Lobulated.or.irregular + Unclear.free.margin + Acute.angle.with.cyst.wall + Proportion.50.+Eccentric.growth, family = binomial, data = train_data)
summary_integrate<-summary(integrate_Chara)
integrate_OR <- exp(coef(integrate_Chara))
integrate_CI <- exp(confint(integrate_Chara))
integrate_result<-cbind(summary_integrate$coef, OR = integrate_OR, `95% CI Lower` = integrate_CI[, 1], `95% CI Upper` = integrate_CI[, 2])
integrate_result

## Estimate Std. Error z value Pr(>|z|)
## (Intercept) -1.03217267 0.7806249 -1.3222389 1.860886e-01
## Microcalcifications 2.03008117 0.5841379 3.4753459 5.101950e-04
## Macrocalcifications 0.20913637 0.7896583 0.2648441 7.911295e-01
## Hypoechoic -0.85836896 0.6214952 -1.3811352 1.672374e-01
## Lobulated.or.irregular 0.26094113 0.6333886 0.4119764 6.803567e-01
## Unclear.free.margin 3.47737122 0.6065960 5.7325984 9.890363e-09
## Acute.angle.with.cyst.wall -0.30836485 0.7679208 -0.4015582 6.880092e-01
## Proportion.50. 2.31514348 0.5443842 4.2527748 2.111379e-05
## Eccentric.growth 0.08461439 0.5209702 0.1624169 8.709775e-01
## OR 95% CI Lower 95% CI Upper
## (Intercept) 0.3562321 0.07487106 1.619548
## Microcalcifications 7.6147044 2.46347625 24.518258
## Macrocalcifications 1.2326131 0.26184875 5.876791
## Hypoechoic 0.4238528 0.12296085 1.418314
## Lobulated.or.irregular 1.2981512 0.37335513 4.549502
## Unclear.free.margin 32.3745046 10.26616249 111.723875
## Acute.angle.with.cyst.wall 0.7346472 0.15979045 3.289415
## Proportion.50. 10.1263758 3.64566030 31.176421
## Eccentric.growth 1.0882973 0.38966358 3.024013

#building model–overall risk characteristics models

library(MASS)
library(pROC)
library(caret)
library(DescTools) # 用于计算置信区间

# 拟合模型
ACR_TIRADS_model <- glm(pathology ~ Hypoechoic + Microcalcifications + Macrocalcifications,
 data = train_data,
 family = binomial)

summary(ACR_TIRADS_model)

##
## Call:
## glm(formula = pathology ~ Hypoechoic + Microcalcifications +
## Macrocalcifications, family = binomial, data = train_data)
##
## Coefficients:
## Estimate Std. Error z value Pr(>|z|)
## (Intercept) -0.8147 0.3439 -2.369 0.01784 *
## Hypoechoic -1.4051 0.5094 -2.758 0.00581 **
## Microcalcifications 2.3692 0.4411 5.371 7.84e-08 ***
## Macrocalcifications -1.3173 0.5445 -2.419 0.01556 *
## ---
## Signif. codes: 0 '***' 0.001 '**' 0.01 '*' 0.05 '.' 0.1 ' ' 1
##
## (Dispersion parameter for binomial family taken to be 1)
##
## Null deviance: 413.68 on 339 degrees of freedom
## Residual deviance: 366.49 on 336 degrees of freedom
## AIC: 374.49
##
## Number of Fisher Scoring iterations: 4

# 提取 OR 和 CI
exp_coef <- exp(coef(ACR_TIRADS_model))
confint_ACR_TIRADS_model <- exp(confint(ACR_TIRADS_model))
result <- cbind(OR = exp_coef, CI_lower = confint_ACR_TIRADS_model[, 1], CI_upper = confint_ACR_TIRADS_model[, 2])
print(result)

## OR CI_lower CI_upper
## (Intercept) 0.4427615 0.22252654 0.8597348
## Hypoechoic 0.2453410 0.08901691 0.6590214
## Microcalcifications 10.6884160 4.59209287 25.9933942
## Macrocalcifications 0.2678553 0.08855297 0.7554793

# 清理数据
train_data_clean <- na.omit(train_data)

# 预测概率
predicted_prob <- predict(ACR_TIRADS_model, newdata = train_data_clean, type = "response")

# ROC曲线和最佳cutoff
roc_curve <- roc(train_data_clean$pathology, predicted_prob)
optimal_cutoff <- coords(roc_curve, "best", best.method = "youden", ret = "threshold")
optimal_cutoff_value <- as.numeric(optimal_cutoff)
cat("Optimal Cut-off value based on Youden's Index:", optimal_cutoff_value, "\n")

## Optimal Cut-off value based on Youden's Index: 0.2569487

# 二分类预测
predicted_class <- ifelse(predicted_prob >= optimal_cutoff_value, 1, 0)

# 混淆矩阵
conf_matrix <- confusionMatrix(factor(predicted_class), factor(train_data_clean$pathology))

# 提取基本性能指标
sensitivity <- conf_matrix$byClass["Sensitivity"]
specificity <- conf_matrix$byClass["Specificity"]
NPV <- conf_matrix$byClass["Neg Pred Value"]
PPV <- conf_matrix$byClass["Pos Pred Value"]
accuracy <- conf_matrix$overall["Accuracy"]

# 获取混淆矩阵
cm_table <- conf_matrix$table
TP <- cm_table["1", "1"] # 预测 1，真实 1
FP <- cm_table["1", "0"] # 预测 1，真实 0
TN <- cm_table["0", "0"] # 预测 0，真实 0
FN <- cm_table["0", "1"] # 预测 0，真实 1

# Sensitivity: 针对真实为 1
sensitivity <- TP / (TP + FN)
# Specificity: 针对真实为 0
specificity <- TN / (TN + FP)
# PPV: 针对预测为 1
PPV <- TP / (TP + FP)
# NPV: 针对预测为 0
NPV <- TN / (TN + FN)
# Accuracy
accuracy <- (TP + TN) / (TP + TN + FP + FN)

# 置信区间计算
sens_ci <- binom.test(TP, TP + FN)$conf.int
spec_ci <- binom.test(TN, TN + FP)$conf.int
ppv_ci <- binom.test(TP, TP + FP)$conf.int
npv_ci <- binom.test(TN, TN + FN)$conf.int
acc_ci <- binom.test(TP + TN, TP + TN + FP + FN)$conf.int
# 输出结果
cat(sprintf("Sensitivity: %.2f (95%% CI: %.2f - %.2f)\n", sensitivity, sens_ci[1], sens_ci[2]))

## Sensitivity: 0.77 (95% CI: 0.68 - 0.85)

cat(sprintf("Specificity: %.2f (95%% CI: %.2f - %.2f)\n", specificity, spec_ci[1], spec_ci[2]))

## Specificity: 0.60 (95% CI: 0.54 - 0.67)

cat(sprintf("Accuracy: %.2f (95%% CI: %.2f - %.2f)\n", accuracy, acc_ci[1], acc_ci[2]))

## Accuracy: 0.65 (95% CI: 0.60 - 0.70)

cat(sprintf("PPV: %.2f (95%% CI: %.2f - %.2f)\n", PPV, ppv_ci[1], ppv_ci[2]))

## PPV: 0.45 (95% CI: 0.38 - 0.53)

cat(sprintf("NPV: %.2f (95%% CI: %.2f - %.2f)\n", NPV, npv_ci[1], npv_ci[2]))

## NPV: 0.86 (95% CI: 0.80 - 0.91)

# AUC 及 95% CI
auc_value <- auc(roc_curve)
auc_ci <- ci.auc(roc_curve)
cat("AUC:", auc_value, "\n")

## AUC: 0.7295248

cat("AUC 95% CI:", auc_ci, "\n")

## AUC 95% CI: 0.6712991 0.7295248 0.7877505

# 模型卡方检验
model_pvalue <- anova(ACR_TIRADS_model, test="Chisq")
cat("Model P-value:", model_pvalue$`Pr(>Chi)`[2], "\n")

## Model P-value: 0.000993799

##building model–Internal risk characteristics models

library(MASS)
library(pROC)
library(caret)

# Internal risk characteristics models
Extra_model <- glm(pathology ~ Unclear.free.margin + Proportion.50.,
 data = train_data,
 family = binomial)

summary(Extra_model)

##
## Call:
## glm(formula = pathology ~ Unclear.free.margin + Proportion.50.,
## family = binomial, data = train_data)
##
## Coefficients:
## Estimate Std. Error z value Pr(>|z|)
## (Intercept) -0.4435 0.4621 -0.960 0.337
## Unclear.free.margin 3.9210 0.4850 8.084 6.27e-16 ***
## Proportion.50. 2.3306 0.5298 4.399 1.09e-05 ***
## ---
## Signif. codes: 0 '***' 0.001 '**' 0.01 '*' 0.05 '.' 0.1 ' ' 1
##
## (Dispersion parameter for binomial family taken to be 1)
##
## Null deviance: 413.68 on 339 degrees of freedom
## Residual deviance: 293.02 on 337 degrees of freedom
## AIC: 299.02
##
## Number of Fisher Scoring iterations: 5

# OR 值和 95% CI
exp_coef <- exp(coef(Extra_model))
confint_Extra_model <- exp(confint(Extra_model))
result <- cbind(OR = exp_coef, CI_lower = confint_Extra_model[, 1], CI_upper = confint_Extra_model[, 2])
print(result)

## OR CI_lower CI_upper
## (Intercept) 0.64179 0.2502646 1.549922
## Unclear.free.margin 50.45091 20.1746674 135.869868
## Proportion.50. 10.28426 3.8080342 30.711741

# 清理数据
train_data_clean <- na.omit(train_data)

# 预测概率
predicted_prob <- predict(Extra_model, newdata = train_data_clean, type = "response")

# ROC曲线和最佳cut-off
roc_curve <- roc(train_data_clean$pathology, predicted_prob)
optimal_cutoff <- coords(roc_curve, "best", best.method = "youden", ret = "threshold")
optimal_cutoff_value <- as.numeric(optimal_cutoff)
cat("Optimal Cut-off value based on Youden's Index:", optimal_cutoff_value, "\n")

## Optimal Cut-off value based on Youden's Index: 0.2994389

# 根据最佳cut-off分类
predicted_class <- ifelse(predicted_prob >= optimal_cutoff_value, 1, 0)

# 混淆矩阵
conf_matrix <- confusionMatrix(factor(predicted_class), factor(train_data_clean$pathology))
cm_table <- conf_matrix$table

# 正确提取 TP, FP, TN, FN
TP <- cm_table["1", "1"]
FP <- cm_table["1", "0"]
TN <- cm_table["0", "0"]
FN <- cm_table["0", "1"]

total <- TP + TN + FP + FN

# 正确计算性能指标
sensitivity <- TP / (TP + FN) # 针对真实为 1
specificity <- TN / (TN + FP) # 针对真实为 0
PPV <- TP / (TP + FP) # 针对预测为 1
NPV <- TN / (TN + FN) # 针对预测为 0
accuracy <- (TP + TN) / total

# 计算置信区间
sens_ci <- binom.test(TP, TP + FN)$conf.int
spec_ci <- binom.test(TN, TN + FP)$conf.int
ppv_ci <- binom.test(TP, TP + FP)$conf.int
npv_ci <- binom.test(TN, TN + FN)$conf.int
acc_ci <- binom.test(TP + TN, total)$conf.int

# 输出结果
cat(sprintf("Sensitivity: %.2f (95%% CI: %.2f - %.2f)\n", sensitivity, sens_ci[1], sens_ci[2]))

## Sensitivity: 0.79 (95% CI: 0.70 - 0.87)

cat(sprintf("Specificity: %.2f (95%% CI: %.2f - %.2f)\n", specificity, spec_ci[1], spec_ci[2]))

## Specificity: 0.80 (95% CI: 0.74 - 0.85)

cat(sprintf("PPV: %.2f (95%% CI: %.2f - %.2f)\n", PPV, ppv_ci[1], ppv_ci[2]))

## PPV: 0.62 (95% CI: 0.54 - 0.71)

cat(sprintf("NPV: %.2f (95%% CI: %.2f - %.2f)\n", NPV, npv_ci[1], npv_ci[2]))

## NPV: 0.90 (95% CI: 0.85 - 0.94)

cat(sprintf("Accuracy: %.2f (95%% CI: %.2f - %.2f)\n", accuracy, acc_ci[1], acc_ci[2]))

## Accuracy: 0.80 (95% CI: 0.75 - 0.84)

# AUC 和 95% CI
auc_value <- auc(roc_curve)
auc_ci <- ci.auc(roc_curve)
cat("AUC:", auc_value, "\n")

## AUC: 0.8489167

cat("AUC 95% CI:", auc_ci, "\n")

## AUC 95% CI: 0.8029967 0.8489167 0.8948367

# 模型整体 P 值
model_pvalue <- anova(Extra_model, test="Chisq")
cat("Model P-value:", model_pvalue$`Pr(>Chi)`[2], "\n")

## Model P-value: 5.777758e-23

##building model–integrated model

###integrated model
library(MASS)
library(pROC)
library(caret)

# Internal risk characteristics models
integrate_model <- glm(pathology ~ Microcalcifications + Unclear.free.margin + Proportion.50.,
 data = train_data,
 family = binomial)

# 输出模型结果
summary(integrate_model)

##
## Call:
## glm(formula = pathology ~ Microcalcifications + Unclear.free.margin +
## Proportion.50., family = binomial, data = train_data)
##
## Coefficients:
## Estimate Std. Error z value Pr(>|z|)
## (Intercept) -1.3845 0.5342 -2.592 0.009556 **
## Microcalcifications 1.9169 0.5330 3.597 0.000322 ***
## Unclear.free.margin 3.7898 0.4983 7.605 2.85e-14 ***
## Proportion.50. 2.2803 0.5266 4.330 1.49e-05 ***
## ---
## Signif. codes: 0 '***' 0.001 '**' 0.01 '*' 0.05 '.' 0.1 ' ' 1
##
## (Dispersion parameter for binomial family taken to be 1)
##
## Null deviance: 413.68 on 339 degrees of freedom
## Residual deviance: 279.38 on 336 degrees of freedom
## AIC: 287.38
##
## Number of Fisher Scoring iterations: 5

# OR值和95%置信区间
exp_coef <- exp(coef(integrate_model))
confint_integrate_model <- exp(confint(integrate_model))
result <- cbind(OR = exp_coef, CI_lower = confint_integrate_model[, 1], CI_upper = confint_integrate_model[, 2])
print(result)

## OR CI_lower CI_upper
## (Intercept) 0.2504481 0.08487515 0.6958767
## Microcalcifications 6.7995345 2.43618987 19.8472608
## Unclear.free.margin 44.2461964 17.24170288 122.4029224
## Proportion.50. 9.7795447 3.64021480 29.0136211

# 清理缺失数据
train_data_clean <- na.omit(train_data)

# 预测概率
predicted_prob <- predict(integrate_model, newdata = train_data_clean, type = "response")

# 计算ROC曲线与最佳cut-off
roc_curve <- roc(train_data_clean$pathology, predicted_prob)
optimal_cutoff <- coords(roc_curve, "best", best.method = "youden", ret = "threshold")
optimal_cutoff_value <- as.numeric(optimal_cutoff)
cat("Optimal Cut-off value based on Youden's Index:", optimal_cutoff_value, "\n")

## Optimal Cut-off value based on Youden's Index: 0.2854971

# 根据cut-off进行分类
predicted_class <- ifelse(predicted_prob >= optimal_cutoff_value, 1, 0)

# 计算混淆矩阵
conf_matrix <- confusionMatrix(factor(predicted_class), factor(train_data_clean$pathology))
cm_table <- conf_matrix$table

# 正确提取TP, FP, TN, FN
TP <- cm_table["1", "1"]
FP <- cm_table["1", "0"]
TN <- cm_table["0", "0"]
FN <- cm_table["0", "1"]

total <- TP + TN + FP + FN

# 正确计算性能指标
sensitivity <- TP / (TP + FN) # 针对真实为 1
specificity <- TN / (TN + FP) # 针对真实为 0
PPV <- TP / (TP + FP) # 针对预测为 1
NPV <- TN / (TN + FN) # 针对预测为 0
accuracy <- (TP + TN) / total

# 计算每个指标的95%置信区间
sens_ci <- binom.test(TP, TP + FN)$conf.int
spec_ci <- binom.test(TN, TN + FP)$conf.int
ppv_ci <- binom.test(TP, TP + FP)$conf.int
npv_ci <- binom.test(TN, TN + FN)$conf.int
acc_ci <- binom.test(TP + TN, total)$conf.int

# 输出带置信区间的性能指标
cat(sprintf("Sensitivity: %.2f (95%% CI: %.2f - %.2f)\n", sensitivity, sens_ci[1], sens_ci[2]))

## Sensitivity: 0.81 (95% CI: 0.72 - 0.88)

cat(sprintf("Specificity: %.2f (95%% CI: %.2f - %.2f)\n", specificity, spec_ci[1], spec_ci[2]))

## Specificity: 0.80 (95% CI: 0.74 - 0.85)

cat(sprintf("PPV: %.2f (95%% CI: %.2f - %.2f)\n", PPV, ppv_ci[1], ppv_ci[2]))

## PPV: 0.63 (95% CI: 0.54 - 0.71)

cat(sprintf("NPV: %.2f (95%% CI: %.2f - %.2f)\n", NPV, npv_ci[1], npv_ci[2]))

## NPV: 0.91 (95% CI: 0.86 - 0.94)

cat(sprintf("Accuracy: %.2f (95%% CI: %.2f - %.2f)\n", accuracy, acc_ci[1], acc_ci[2]))

## Accuracy: 0.80 (95% CI: 0.76 - 0.84)

# 计算AUC及95%置信区间
auc_value <- auc(roc_curve)
auc_ci <- ci.auc(roc_curve)
cat("AUC:", auc_value, "\n")

## AUC: 0.8624218

cat("AUC 95% CI:", auc_ci, "\n")

## AUC 95% CI: 0.8182177 0.8624218 0.9066259

# 计算模型卡方检验P值
model_pvalue <- anova(integrate_model, test = "Chisq")
cat("Model P-value:", model_pvalue$`Pr(>Chi)`[2], "\n")

## Model P-value: 4.410646e-07

library(pROC)
train_data_clean <- na.omit(train_data)
# 预测概率
pred_prob_ACR_TIRADS <- predict(ACR_TIRADS_model, newdata = train_data_clean, type = "response")
pred_prob_Extra <- predict(Extra_model, newdata = train_data_clean, type = "response")
pred_prob_Integrated <- predict(integrate_model, newdata = train_data_clean, type = "response")
# 绘制ROC曲线，增加美化效果
roc_ACR_TIRADS <- roc(train_data_clean$pathology, pred_prob_ACR_TIRADS)
roc_Extra <- roc(train_data_clean$pathology, pred_prob_Extra)
roc_Integrated <- roc(train_data_clean$pathology, pred_prob_Integrated)
# 设置图形参数
plot(roc_ACR_TIRADS, col = "#1f78b4", lwd = 2, main = "Comparative ROC Curves for Models",
 xlim = c(1, 0), ylim = c(0, 1), grid = TRUE, legacy.axes = TRUE)
plot(roc_Extra, col = "#33a02c", lwd = 2, add = TRUE)
plot(roc_Integrated, col = "#e31a1c", lwd = 2, add = TRUE)
# 添加网格线
grid()
# 添加图例，设置为美观的透明背景
legend("bottomright", legend = c("Overall risk characteristics of entire nodule model", "Internal risk characteristics of the solid part model", "Integrated risk characteristic model"),
 col = c("#1f78b4", "#33a02c", "#e31a1c"), lwd = 2, box.lty = 0, bg = 'transparent')


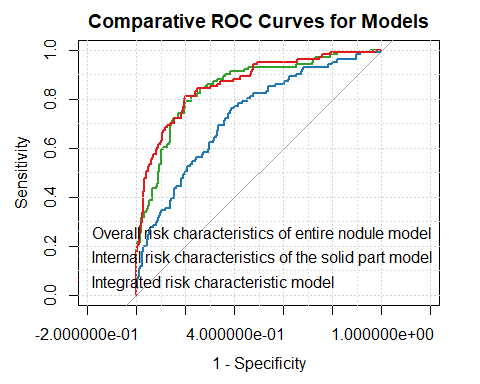


# 输出AUC值
cat("AUC for ACR_TIRADS_model:", auc(roc_ACR_TIRADS), "\n")

## AUC for ACR_TIRADS_model: 0.7295248

cat("AUC for Extra_model:", auc(roc_Extra), "\n")

## AUC for Extra_model: 0.8489167

cat("AUC for Integrate_model:", auc(roc_Integrated), "\n")

## AUC for Integrate_model: 0.8624218

#delong test in training set
roc_ACR_TIRADS <- roc(train_data_clean$pathology, pred_prob_ACR_TIRADS)
roc_Extra <- roc(train_data_clean$pathology, pred_prob_Extra)
roc_Integrated <- roc(train_data_clean$pathology, pred_prob_Integrated)
# 比较ACR_TIRADS_model和Extra_model的AUC
delong_test_1 <- roc.test(roc_ACR_TIRADS, roc_Extra, method = "delong")
cat("P-value for DeLong test between ACR_TIRADS_model and Extra_model:", delong_test_1$p.value, "\n")

## P-value for DeLong test between ACR_TIRADS_model and Extra_model: 0.0002300151

# 比较ACR_TIRADS_model和Integrated_model的AUC
delong_test_2 <- roc.test(roc_ACR_TIRADS, roc_Integrated, method = "delong")
cat("P-value for DeLong test between ACR_TIRADS_model and Integrated_model:", delong_test_2$p.value, "\n")

## P-value for DeLong test between ACR_TIRADS_model and Integrated_model: 2.353639e-06

# 比较Extra_model和Integrated_model的AUC
delong_test_3 <- roc.test(roc_Extra, roc_Integrated, method = "delong")
cat("P-value for DeLong test between Extra_model and Integrated_model:", delong_test_3$p.value, "\n")

## P-value for DeLong test between Extra_model and Integrated_model: 0.1008072

###validation in testing dataset

library(pROC)
library(caret)

# 定义性能指标函数
evaluate_test_performance <- function(predicted_prob, true_labels, cutoff_value) {
 # 分类
 predicted_class <- ifelse(predicted_prob >= cutoff_value, 1, 0)

 # 混淆矩阵
 conf_matrix <- confusionMatrix(factor(predicted_class), factor(true_labels))
 cm_table <- conf_matrix$table

 # 提取 TP, TN, FP, FN
 TP <- cm_table["1", "1"]
 FP <- cm_table["1", "0"]
 TN <- cm_table["0", "0"]
 FN <- cm_table["0", "1"]
 total <- TP + TN + FP + FN

 # 指标计算
 sensitivity <- TP / (TP + FN)
 specificity <- TN / (TN + FP)
 PPV <- TP / (TP + FP)
 NPV <- TN / (TN + FN)
 accuracy <- (TP + TN) / total

 # 置信区间
 sens_ci <- binom.test(TP, TP + FN)$conf.int
 spec_ci <- binom.test(TN, TN + FP)$conf.int
 ppv_ci <- binom.test(TP, TP + FP)$conf.int
 npv_ci <- binom.test(TN, TN + FN)$conf.int
 acc_ci <- binom.test(TP + TN, total)$conf.int

 # 输出结果
 cat(sprintf("Sensitivity: %.2f (95%% CI: %.2f - %.2f)\n", sensitivity, sens_ci[1], sens_ci[2]))
 cat(sprintf("Specificity: %.2f (95%% CI: %.2f - %.2f)\n", specificity, spec_ci[1], spec_ci[2]))
 cat(sprintf("PPV: %.2f (95%% CI: %.2f - %.2f)\n", PPV, ppv_ci[1], ppv_ci[2]))
 cat(sprintf("NPV: %.2f (95%% CI: %.2f - %.2f)\n", NPV, npv_ci[1], npv_ci[2]))
 cat(sprintf("Accuracy: %.2f (95%% CI: %.2f - %.2f)\n\n", accuracy, acc_ci[1], acc_ci[2]))
}

# 读取数据
library(xlsx)
test_data <- read.xlsx("E:/R/PCTN/486 ML model/Testing data.xlsx", 1)
test_data_clean <- na.omit(test_data)

# 取反
test_data_clean$Unclear.free.margin <- -test_data_clean$Unclear.free.margin
test_data_clean$Lobulated.or.irregular <- -test_data_clean$Lobulated.or.irregular

# 三个模型预测概率
pred_prob_ACR_TIRADS_test <- predict(ACR_TIRADS_model, newdata = test_data_clean, type = "response")
pred_prob_Extra_test <- predict(Extra_model, newdata = test_data_clean, type = "response")
pred_prob_Integrated_test <- predict(integrate_model, newdata = test_data_clean, type = "response")

# ROC 曲线 & 最佳 cut-off
roc_ACR_TIRADS_test <- roc(test_data_clean$pathology, pred_prob_ACR_TIRADS_test)
roc_Extra_test <- roc(test_data_clean$pathology, pred_prob_Extra_test)
roc_Integrated_test <- roc(test_data_clean$pathology, pred_prob_Integrated_test)

# AUC 及 95% CI
cat("AUC for ACR_TIRADS_model on Test Data:", auc(roc_ACR_TIRADS_test), "\n")

## AUC for ACR_TIRADS_model on Test Data: 0.8453256

cat("AUC 95% CI:", ci.auc(roc_ACR_TIRADS_test), "\n\n")

## AUC 95% CI: 0.7742306 0.8453256 0.9164206

cat("AUC for Extra_model on Test Data:", auc(roc_Extra_test), "\n")

## AUC for Extra_model on Test Data: 0.9319853

cat("AUC 95% CI:", ci.auc(roc_Extra_test), "\n\n")

## AUC 95% CI: 0.8833677 0.9319853 0.9806029

cat("AUC for Integrated_model on Test Data:", auc(roc_Integrated_test), "\n")

## AUC for Integrated_model on Test Data: 0.9606092

cat("AUC 95% CI:", ci.auc(roc_Integrated_test), "\n\n")

## AUC 95% CI: 0.9316401 0.9606092 0.9895783

# 最佳 cut-off
optimal_cutoff_ACR_TIRADS <- as.numeric(coords(roc_ACR_TIRADS_test, "best", best.method = "youden", ret = "threshold"))
optimal_cutoff_Extra <- as.numeric(coords(roc_Extra_test, "best", best.method = "youden", ret = "threshold"))
optimal_cutoff_Integrated <- as.numeric(coords(roc_Integrated_test, "best", best.method = "youden", ret = "threshold"))

cat("Optimal Cut-off for ACR_TIRADS_model on Test Data:", optimal_cutoff_ACR_TIRADS, "\n")

## Optimal Cut-off for ACR_TIRADS_model on Test Data: 0.323852

cat("Optimal Cut-off for Extra_model on Test Data:", optimal_cutoff_Extra, "\n")

## Optimal Cut-off for Extra_model on Test Data: 0.4820481

cat("Optimal Cut-off for Integrated_model on Test Data:", optimal_cutoff_Integrated, "\n\n")

## Optimal Cut-off for Integrated_model on Test Data: 0.4147123

# 分别评估三个模型
cat("Performance metrics for ACR_TIRADS_model on Test Data:\n")

## Performance metrics for ACR_TIRADS_model on Test Data:

evaluate_test_performance(pred_prob_ACR_TIRADS_test, test_data_clean$pathology, optimal_cutoff_ACR_TIRADS)

## Sensitivity: 0.76 (95% CI: 0.59 - 0.89)
## Specificity: 0.75 (95% CI: 0.66 - 0.83)
## PPV: 0.48 (95% CI: 0.34 - 0.62)
## NPV: 0.91 (95% CI: 0.84 - 0.96)
## Accuracy: 0.75 (95% CI: 0.68 - 0.82)

cat("Performance metrics for Extra_model on Test Data:\n")

## Performance metrics for Extra_model on Test Data:

evaluate_test_performance(pred_prob_Extra_test, test_data_clean$pathology, optimal_cutoff_Extra)

## Sensitivity: 0.85 (95% CI: 0.69 - 0.95)
## Specificity: 0.92 (95% CI: 0.85 - 0.96)
## PPV: 0.76 (95% CI: 0.60 - 0.89)
## NPV: 0.95 (95% CI: 0.90 - 0.98)
## Accuracy: 0.90 (95% CI: 0.84 - 0.95)

cat("Performance metrics for Integrated_model on Test Data:\n")

## Performance metrics for Integrated_model on Test Data:

evaluate_test_performance(pred_prob_Integrated_test, test_data_clean$pathology, optimal_cutoff_Integrated)

## Sensitivity: 0.88 (95% CI: 0.73 - 0.97)
## Specificity: 0.92 (95% CI: 0.85 - 0.96)
## PPV: 0.77 (95% CI: 0.61 - 0.89)
## NPV: 0.96 (95% CI: 0.91 - 0.99)
## Accuracy: 0.91 (95% CI: 0.85 - 0.95)

##绘验证集的图
roc_ACR_TIRADS_test <- roc(test_data_clean$pathology, pred_prob_ACR_TIRADS_test)
roc_Extra_test <- roc(test_data_clean$pathology, pred_prob_Extra_test)
roc_Integrated_test <- roc(test_data_clean$pathology, pred_prob_Integrated_test)

# 设置图形参数
plot(roc_ACR_TIRADS_test, col = "#1f78b4", lwd = 2, main = "Comparative ROC Curves for Test Data",
 xlim = c(1, 0), ylim = c(0, 1), grid = TRUE, legacy.axes = TRUE)
plot(roc_Extra_test, col = "#33a02c", lwd = 2, add = TRUE)
plot(roc_Integrated_test, col = "#e31a1c", lwd = 2, add = TRUE)

# 添加网格线
grid()

# 添加图例，设置为美观的透明背景
legend("bottomright", legend = c("Overall risk characteristics of entire nodule model", "Internal risk characteristics of the solid part model", "Integrated risk characteristic model"),
 col = c("#1f78b4", "#33a02c", "#e31a1c"), lwd = 2, box.lty = 0, bg = 'transparent')


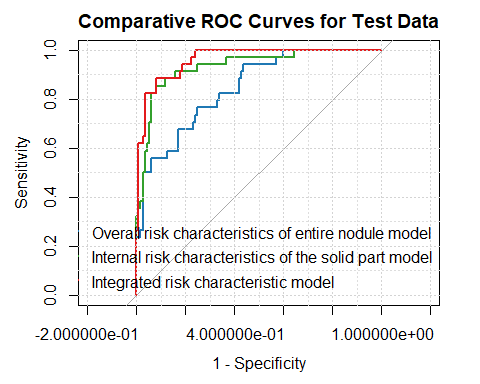


# 输出AUC值
cat("AUC for ACR_TIRADS_model on Test Data:", auc(roc_ACR_TIRADS_test), "\n")

## AUC for ACR_TIRADS_model on Test Data: 0.8453256

cat("AUC for Extra_model on Test Data:", auc(roc_Extra_test), "\n")

## AUC for Extra_model on Test Data: 0.9319853

cat("AUC for Integrate_model on Test Data:", auc(roc_Integrated_test), "\n")

## AUC for Integrate_model on Test Data: 0.9606092
